# Chili Intake Is Inversely Associated with Chronic Kidney Disease among Adults: A Population-Based Study

**DOI:** 10.3390/nu11122949

**Published:** 2019-12-04

**Authors:** Zumin Shi, Ming Zhang, Jianghong Liu

**Affiliations:** 1 Human Nutrition Department, College of Health Sciences, QU Health, Qatar University, Doha 2713, Qatar; 2 Department of Nutrition, Peking University Shenzhen Hospital, Shenzhen 518036, China; zm1633@163.com; 3 University of Pennsylvania School of Nursing, Philadelphia, PA 19104, USA; jhliu@nursing.upenn.edu

**Keywords:** chili intake, chronic kidney disease, Chinese, adults

## Abstract

We aimed to assess the association between chili consumption and kidney function and chronic kidney disease (CKD). Data from 8429 adults attending the China Health and Nutrition Survey were used. Chili intake was assessed using a 3 day, 24 h food record in combination with household food inventory between 1991 and 2009. CKD was defined as an estimated glomerular filtration rate (eGFR) of <60 mL/min/1.73 m^2^, as measured in 2009. Logistic regression was used to assess the association. Of the 8429 participants, 1008 (12.0%) fit the definition of CKD. The prevalence of CKD was 13.1% in non-consumers of chili and 7.4% among those with chili intake above 50 g/day. After adjusting for demographics, lifestyle factors (i.e., smoking, alcohol drinking, physical activity), dietary patterns, and chronic conditions, the odds ratio (OR) (95% CI) for CKD across chili consumption levels of none, 1–20 g/day, 20.1–50 g/day, ≥50.1 g/day were 1.00 (reference), 0.82 (0.67–1.01), 0.83 (0.65–1.05), and 0.51 (0.35–0.75), respectively (*p* for trend 0.001). There was no interaction between chili intake with gender, income, urbanization, hypertension, obesity, or diabetes. This longitudinal large population-based study suggests that chili consumption is inversely associated with CKD, independent of lifestyle, hypertension, obesity, and overall dietary patterns.

## 1. Introduction

Chronic kidney disease (CKD) is a global health issue contributing to many adverse health outcomes and economic burden [1]. It is a common chronic condition affecting 13.4% of the general population globally and 9.5% in China [1]. In addition to the known risk factors including hypertension, diabetes, obesity, and use of nephrotoxic medication [2], individual nutrients and foods [3,4,5,6] as well as overall dietary patterns and food preference [7,8,9] play an important role in the development and management of CKD. 

We have previously reported that a traditional dietary pattern characterized by high intake of rice, pork, and vegetables is positively associated with CKD among participants of the China Health and Nutrition Survey (CHNS) [10]. While total vegetable intake was positively associated with CKD, the association between the specific types of vegetables and CKD has not been examined [10]. There have been mixed findings on the association between vegetable consumption and risk of CKD. Studies showing a positive relationship may be due to contaminants and heavy metals present in those vegetables [10], or due to food preparation. On the other hand, studies have shown a negative relationship, which argues that nutrients from vegetables serve as a protective factor for CKD [11]. We aim to examine chili as a vegetable in relation to CKD.

Chili is one of the most commonly used spices in the world [12], especially in Asia [13]. In China, residents in some regions (e.g., Sichuan, Hunan) have a high consumption of chili [14]. In these regions, chili is not only used as a spice but also as a vegetable. The large variation of chili consumption in China allows for the use of an epidemiological approach to the study of the effects of chili consumption on health outcomes. Recently, an increasing number of studies have identified both beneficial and adverse effects. It has been found that chili consumption is inversely related to mortality [14], obesity [15], and hypertension [16], but positively associated with poor cognition [17]. The binding of capsaicin, the active component of chili, to its receptor leads to inhibition of vascular oxidative stress [18], reduced energy intake, increased energy expenditure, and enhanced fat oxidation [19,20,21,22,23]. These effects of capsaicin have been hypothesized to be responsible for beneficial effects of chili on body weight and blood pressure. 

In animal models, it has been shown that capsaicin has beneficial effects on kidney function through acting as a diuretic in healthy and diabetic rats [24] and reducing urinary epidermal growth factor (EGF) levels, a biomarker for kidney disease [24]. Activation of the capsaicin receptor TRPV1 can prevent salt-induced kidney damage in rats [25] and increase glomerular filtration rate (GFR) and renal excretion by increasing natriuresis and diuresis [26,27], indications of kidney function. Thus, current evidence from animal studies suggests a potential role of capsaicin in maintaining kidney function. However, this assertion has not been investigated in humans, and no population studies have assessed the association between chili consumption and CKD. Chili intake has been shown to be inversely associated with the risk of obesity [15] and hypertension [16] in the Chinese population. Compared with non-consumers, those with chili intake above 50 g/day had 27% and 35% reduced risk of developing obesity and hypertension, respectively. This study aimed to assess the association between chili intake and CKD among Chinese adults who participated in the Chinese Health and Nutrition Survey (CHNS).

Furthermore, chronic illnesses such as obesity, hypertension, and diabetes are risk factors for kidney disease. It is possible that the beneficial effects of chili on these conditions contribute to the reduction of kidney disease, rather than the direct effect of chili consumption on kidney function. Therefore, our second aim is to assess whether chili is independently associated with CKD by controlling for a variety of confounding factors as well as testing for any interaction effects.

## 2. Materials and Methods 

### 2.1. Study Design and Sample

**Figure 1:**
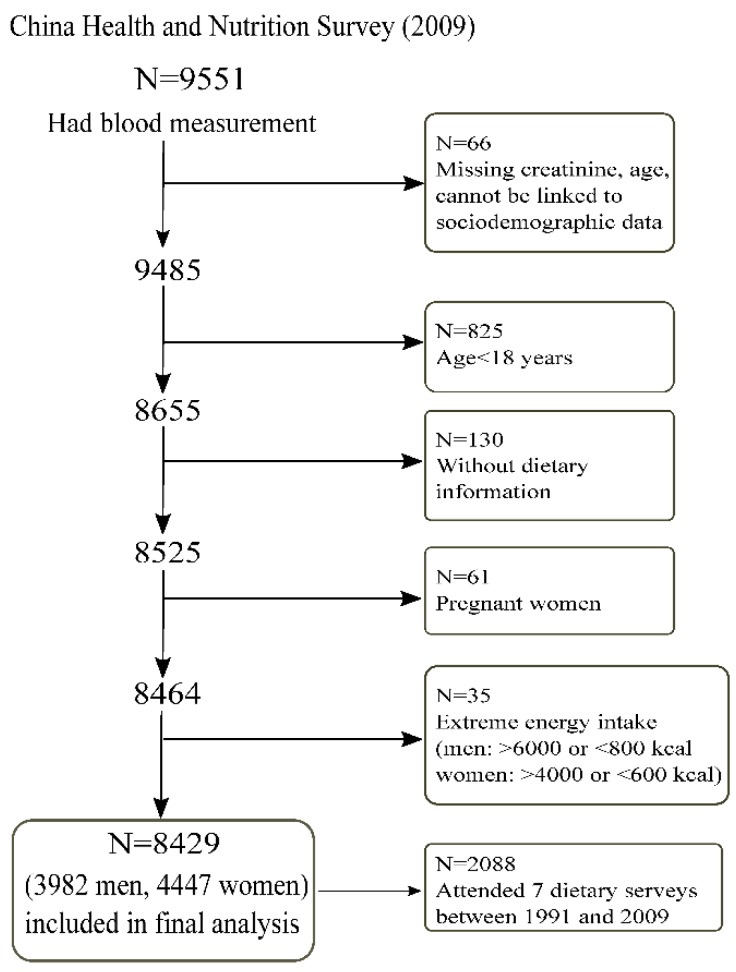
Sample flowchart of participants attending the China Health and Nutrition Survey in 2009.

The CHNS study is an ongoing, open prospective, cohort study that used a multistage random-cluster sampling method to select participants from nine provinces in China from 1989 to 2015. There have been ten waves of data collection (1989, 1991, 1993, 1997, 2000, 2004, 2006, 2009, 2011, and 2015) thus far. Dietary intake was assessed by inviting all members in the selected household to complete each survey between 1991 and 2009. Blood samples were collected at the study sites in the 2009 and 2015 surveys and shipped to Beijing for measurements including serum creatinine. However, the blood measurements in the 2015 survey are not available currently. In the 2009 survey, a total of 9551 out of the 18,887 (50.6%) participants had blood measurements to assess renal function (Figure 1). In the currently analysis, we excluded those below 18 years of age, those who did not provide dietary information in 2009, those who were pregnant, and those who had an extreme intake of energy (i.e., men: >6000 or <800 kcal; women: >4000 or <600 kcal). A total of 8429 participants met the inclusion criteria and were included in the final analytical sample. Informed consent was obtained from all participants, and ethical approval was granted by the institutional review committees of the University of North Carolina and the National Institute of Nutrition and Food Safety.

### 2.2. Outcome Variable: Estimated GFR and CKD

Kidney function was evaluated by the estimated glomerular filtration rate (eGFR) using serum creatinine. Serum creatinine was measured using Jaffe’s kinetic method (Hitachi 7600 automated analyzer, Hitachi Inc., Tokyo, Japan). The CKD-EPI creatinine equation was used to estimate eGFR [28]. CKD was defined as eGFR <60 mL/min/1.73 m^2^ [29].

### 2.3. Exposure Variable: Cumulative Mean Chili Intake

Chili intake, including both fresh and dried chili peppers (Figure S1), was derived from dietary surveys between 1991 and 2009. In the analysis, chili intake did not include sweet capsicum or black pepper. It was calculated from a cumulative average for each individual at each survey from all the proceeding chili intakes in order to reduce variation within individuals. The use of cumulative average intake from different surveys represented long term habitual intake [30]. The chili intake for mixed dishes was estimated based on the fresh ingredients. In the analysis, the cumulative mean chili intake was recoded into four groups: non-consumers, 1–20 g/day, 20.1–50 g/day, and ≥50.1 g/day. The use of the cut-off was based on our previous paper as well as the serving size [16]. The serving size in the context of Chinese food is a *Liang* (1 *Liang* = 50 g). The median portion size of chili intake was 50 g. Furthermore, among the chili consumers, about 30% had chili intake above 50 g per day [16].

The details of the dietary survey has been published elsewhere [31]. All foods and condiments in the home inventory, purchased from shops/markets or picked from home gardens, and food waste, were weighed and recorded by interviewers during the three-day food consumption survey. Individual dietary intake data were collected by a trained investigator on each of three consecutive days. Food consumption data were analyzed and converted into nutrient intake using the Chinese Food Composition Table. The dietary assessment method has been validated for energy intake [32].

### 2.4. Covariates

A structured questionnaire was used in 2009 during home interviews to collect information on sociodemographic and lifestyle factors. Physical activity level (metabolic equivalent of task, (MET)) was estimated based on self-reported leisure time, occupational, domestic, and commuting activities, and duration using a Compendium of Physical Activities. Smoking status was based on self-report and categorized as non-smokers, ex-smokers, and current smokers. Residence was assessed by a twelve-component urbanization index and recoded into tertiles, which aims to capture population density and physical, social, cultural, and economic environments [31]. Education was categorized as low (illiterate/primary school), medium (junior middle school), and high (high middle school or higher). Per capita annual family income was recoded into tertiles (low, medium, and high). Blood samples collected in 2009 were tested for fasting plasma glucose (FPG) and glycated hemoglobin (HbA1c) [33]. Diabetes was defined as FPG ≥7.0 mmol/L, HbA1C ≥6.5 or having known diabetes (self-reported doctor diagnosed) [34]. C-reactive protein (CRP) in 2009 was measured in blood via the immunoturbidimetric method with Denka Seiken, Japan reagents. We define hypertension as systolic blood pressure above 140 mmHg and/or diastolic blood pressure above 90 mmHg, or having known hypertension [35]. Overweight was defined as BMI ≥25 kg/m^2^ according to the World Health Organization cut-off.

Two dietary patterns (traditional south pattern and modern pattern) were constructed using factor analysis based on our previous publication [10]. The traditional south pattern is characterized by a high intake of rice, pork, and vegetables, and a low intake of wheat; a modern dietary pattern has a high intake of fruits, soy milk, eggs, milk, and deep fried products.

### 2.5. Data Analyses 

Sample characteristics were presented as mean (SD) or percentage. Multivariable logistic regression was used to assess the association between cumulative chili intake between 1991 and 2009 with CKD in 2009. Three multivariable logistic regression models were used: Model 1 adjusted for age in 2009, gender, and energy intake; Model 2 further adjusted for education (low, medium, high), income (tertiles), urbanization level (tertiles), physical activity, smoking (non-smoker, ex-smoker, current smoker), alcohol drinking, and dietary patterns (mean score between 1991 and 2009); Model 3 further adjusted for overweight/obesity (yes or no), hypertension (yes or no), and diabetes (yes or no). The variables adjusted in the logistic models were either known risk factors of CKD or sociodemographic factors (i.e., income, education, urbanization levels). Based on our previous study, dietary patterns were associated with CKD in the Chinese population. As chili intake was strongly associated with dietary patterns, we adjusted for mean dietary pattern scores between 1991 and 2009 as confounding factors. In a sensitivity analysis, we included those subjects who attended all seven waves of dietary measurements (N = 2088). The association between chili intake and eGFR was assessed in a multivariable linear regression model with the adjustment for the same covariates as Model 3 described above among those who attended all seven waves (between 1991 and 2009) of dietary measurements. Marginal means were calculated with the *margins* command and visually presented using the *marginsplot* command in STATA. We tested the multiplicative interaction between sociodemographic factors (i.e., gender, income, urbanization level), hypertension, overweight, diabetes, and chili intake by adding a cross-product term in the main multivariable model (Model 3). To test the non-linear association between chili intake and CKD, we put a linear and a quadratic term of chili intake in a multivariable logistic regression model. All the analyses were performed by using STATA 16 (Stata Corporation, College Station, TX, USA). Statistical significance was considered when *p* < 0.05 (two sided). 

## 3. Results

### 3.1. Sample Characteristics

The mean age of the 8429 participants (3982 men and 4447 women) in the analytical sample was 51.0 (SD 15.0) years in 2009. Among the participants, the median number of dietary measurements was five (interquartile range 2–7). In total, 2088 (24.8%) participants attended all the seven surveys between 1991 and 2009. The cumulative mean intake of chili was 15.9 g/day (SD 23.1). Of the participants, 689 (8.2%) had chili intake greater than 50 g/day. The mean eGFR in 2009 was 79.1 mL/min/1.73 m^2^ (SD 16.8), and 1008 (12.0%) of the participants had a CKD based on eGFR of < 60 mL/min/1.73 m^2^. 

Across the levels of cumulative mean chili intake levels from low to high, the intake of energy, fat, protein, and carbohydrate increased (Table 1). High chili consumers were younger and had a lower BMI and a lower prevalence of hypertension and diabetes. Chili intake was positively associated with the traditional dietary pattern, but inversely associated with the modern dietary pattern.

### 3.2. Association Between Chili Intake and CKD

The prevalence of CKD across chili consumption levels of none, 1–20 g/day, 20.1–50 g/day, and ≥50.1 g/day were 13.1%, 11.7%, 11.9%, and 7.4%, respectively. After adjusting for sociodemographic, dietary patterns, and lifestyle factors as well as overweight/obesity, hypertension, and diabetes, the odds ratio (OR) (95% CI) for CKD across chili consumption levels of none, 1–20 g/day, 20.1–50 g/day, and ≥50.1 g/day were 1.00 (reference), 0.82 (0.67–1.01), 0.83 (0.65–1.05), and 0.51 (0.35–0.75), respectively (*p* for trend 0.001) (Table 2). The association remained when the analysis was limited to those with all seven waves of dietary intake (N = 2088) with corresponding ORs (95% CI) for CKD of 1.00, 0.78 (0.54–1.11), 0.73 (0.49–1.08), and 0.47 (0.26–0.85) (*p* for trend 0.012) across the levels of chili intake. Excluding those with a self-reported history of cardiovascular disease did not change the findings (data not shown). 

**Figure 2:**
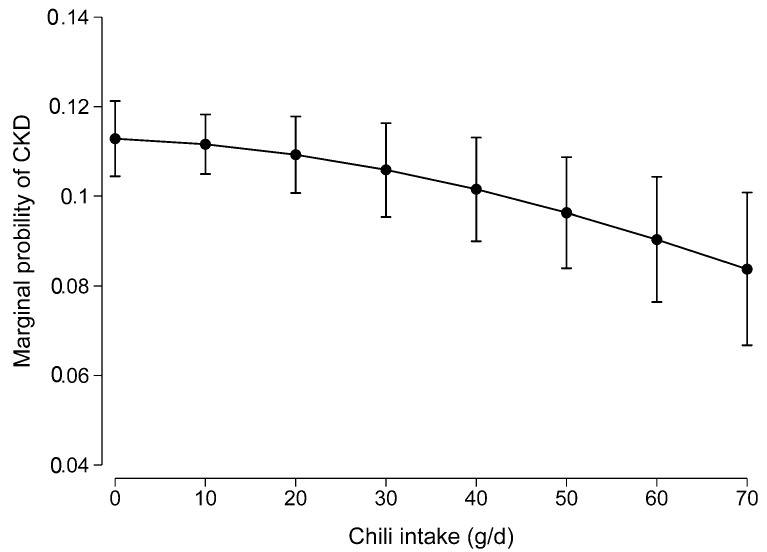
Non-linear association between chili intake and CKD. Values were the marginal probability of CKD derived from a multivariable logistic regression model adjusted for age, gender, intake of energy, education, income, urbanization level, smoking, alcohol drinking, physical activity, dietary patterns, overweight/obesity, hypertension, and diabetes. The *p* for the quadratic term of chili intake was 0.307 in the model.

There was no non-linear association between chili intake and CKD (*p* for the quadratic term of chili intake was 0.307) (Figure 2). 

**Figure 3:**
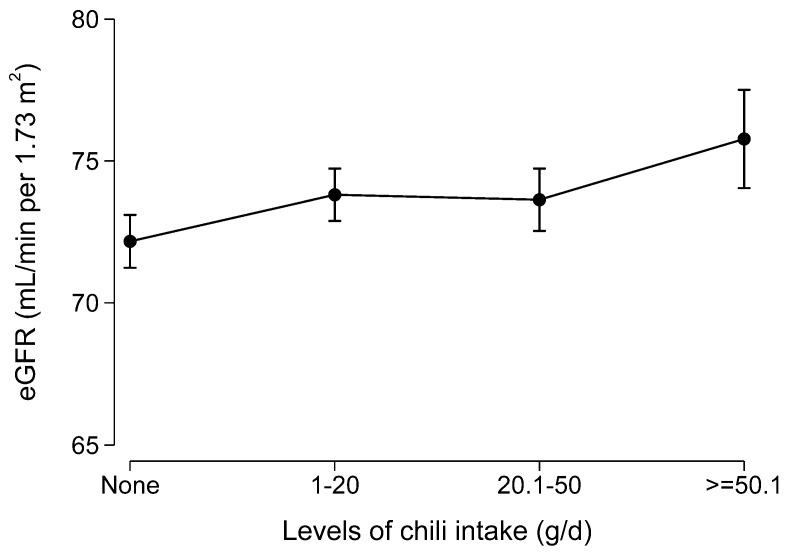
Marginal means of estimated glomerular filtration rate (eGFR) by levels of chili intake among participants who attended all seven waves of the dietary survey (N = 2088). Values were marginal means derived from a multivariable regression model adjusted for age, gender, intake of energy, education, income, and urbanization level.

Among the 2088 participants who attended all the seven surveys, cumulative chili intake was positively associated with eGFR after adjusting for sociodemographic and lifestyle factors, and hypertension, BMI, and diabetes (Figure 3). 

### 3.3. Subgroup Analyses by Sociographic Factors and Chronic Conditions 

No interactions between chili intake with gender, income, urbanization, BMI, hypertension, and diabetes were found (Table 3). The association between high chili intake and CKD was quite similar across genders, income, overweight, hypertension, and diabetes status. 

## 4. Discussion

In this large population-based study, high chili intake was inversely associated with CKD as measured by estimated glomerular filtration rate among Chinese adults who participated in the CHNS between 1991 and 2009. The association was independent of lifestyle factors, dietary patterns, BMI, and hypertension, suggesting a potential direct positive role of chili consumption on kidney function. The positive association between chili intake and kidney function showed a dose-response manner and was independent of lifestyle factors and chronic conditions. The strength of the association between chili intake and CKD was similar in both genders as well as in different demographic backgrounds (e.g., income and urbanization). To our knowledge, this is the first population-based study to investigate the association between chili intake and CKD.

In our previous studies, we found chili intake decreased the risk of obesity [15] and hypertension [16]. In a study conducted in China, Li et al. showed that enjoyment of spicy taste enhanced the sensitivity to salty taste and lowered the daily salt intake and blood pressure [36]. Adding capsaicin to the diet can increase the sensation of fullness and reduce energy intake [37]. In the current study, the effect of chili on CKD appears to be similar regardless of BMI and blood pressure status. It is thus less likely that the association between chili intake and CKD is a result of reduced BMI and hypertension due to chili consumption. In China, diabetes is one of the main contributors to CKD [2,38]. This current study also suggests the inverse association between chili intake and diabetes. Therefore, it could be possible that diabetes contributes to the association between chili intake and CKD. However, in our analysis, the strength of the association between chili and CKD was similar among those with or without diabetes and suggests the relationship between chili consumption and CKD is independent of diabetes. 

Chili consumption may represent a different dietary habit or lifestyle. The association between chili intake and CKD could be confounded by other dietary intakes. For example, individuals who consume more chili are more likely to have a higher intake of cooking oil due to the use of the stir frying cooking method or to have hot pot dishes (*Huoguo* in Chinese). In urban settings, this may represent high consumption of animal foods. In our study, chili was positively associated with a traditional dietary pattern (high intake of rice, vegetables, and pork) but inversely associated with a modern pattern (Table 1). In our previous study, the intake of a traditional dietary pattern was positively associated with CKD, while a modern pattern was inversely associated with CKD [10]. Thus, it is unlikely that the inverse association between chili and CKD is due to dietary patterns. Results from multivariable logistic regression models with and without the adjustment for dietary patterns suggest that the association between chili intake and CKD was independent of dietary patterns. Similarly, many lifestyle factors contribute to kidney function. For example, physical activity and exercise have been positively linked with proper kidney function [39], while physical inactivity is associated with increased mortality in CKD and non-CKD populations [40]. Our findings from multivariable logistic regression models with and without adjustment for physical activity highlight the independent contribution of chili intake on CKD. Furthermore, although chili intake was associated with sociodemographic factors, stratification analyses did not find any interaction between these factors with chili intake.

The mechanisms linking chili intake and kidney function have yet to be fully elucidated. Evidence from animal studies provide support for a beneficial effect of capsaicin in kidney function. Jung et al. reported that capsaicin treatment could ameliorate renal injury through induction of heme oxygenase-1 (HO-1) as well as reduce inflammation and oxidative stress in animal models of cisplatin-induced renal toxicity [41]. In the experiment, the protective effects of capsaicin on cisplatin-induced cell death could be reversed by pharmacological inhibition or knockdown of HO-1. Additionally, activation of TRPV1 (transient receptor potential vanilloid subtype 1, a capsaicin receptor) by capsaicin prevents salt-induced kidney damage and hypertension after renal ischemia-reperfusion injury in rats [25]. An 8 week study conducted by Rios-Silva et al. suggested that capsaicin has a diuretic effect in healthy and diabetic male Wistar rats and reduces the urinary EGF levels [24]. Putting this together, capsaicin may serve to reverse kidney damage and act as a protective factor against renal injury. 

Our study has several strengths. Firstly, we have a relatively large sample size across nine provinces in China as well as multiple measures of chili intake. The food cultures in these provinces are different and provide a wide variation of chili consumption. It provides a unique opportunity to examine the association between chili intake and CKD. The use of cumulative mean chili intake between 1991 and 2009 based on the repeated measure of 3 day dietary intake in combination with household food inventory provides a robust estimate of long-term chili intake. Secondly, it is based on an established ongoing cohort study. We were able to adjust for a variety confounding factors. The main limitation of the study is its cross-sectional analysis. Kidney function was only measured in 2009. Causation cannot be established. It is well recognized by the public that based on the traditional Chinese medicine people with certain chronic diseases should limit their chili consumption. Thus, a potential converse association is possible. However, when we limit our analysis to those without hypertension or obesity, the inverse association between chili intake and CKD remained. As chili consumption is inversely associated with age, although we have adjusted for age and other sociodemographic factors, residual confounding effects may still exist. Furthermore, the statistical analyses did not take the multistage cluster sampling into account due to the complex nature of the open cohort study design. Thus, there may be an increased probability of a Type I error. On the other hand, we adjusted for the residence (i.e. urbanization level), which could partly address the clustering sampling method. In addition, future randomized control trials are needed to confirm the relationship to confirm chili intake and kidney function.

In conclusion, high chili intake was inversely associated with CKD among Chinese adults. The association was independent of hypertension, BMI, and overall dietary patterns. Given that chili is a highly common spice used around the world, more research is warranted to further replicate findings and understand causal mechanisms of the relationship between chili intake and CKD before clinical recommendations and implications can be implemented.

## Figures and Tables

**Table 1:** Sample characteristics in 2009 by levels of cumulative mean chili intake (N = 8429) ^1^. CRP: C-reactive protein; CKD: chronic kidney disease; MET: metabolic equivalent of task.

	None	1–20 g/day	20.1–50 g/day	≥50.1 g/day	*p*-Value
N	3390	2617	1733	689	
Chili intake (g/day), mean (SD)	0.0 (0.0)	9.7 (5.6)	32.8 (8.4)	74.5 (25.8)	<0.001
Traditional dietary pattern, mean (SD)	−0.1 (0.9)	−0.0 (0.8)	0.2 (0.7)	0.4 (0.8)	<0.001
Modern dietary pattern, mean (SD)	0.4 (1.0)	0.2 (0.8)	0.0 (0.8)	−0.1 (0.7)	<0.001
Energy intake (kcal/day), mean (SD)	2074.1 (610.7)	2117.2 (648.7)	2196.0 (630.5)	2310.8 (699.8)	<0.001
Fat intake (g/day), mean (SD)	71.8 (33.1)	74.1 (35.9)	77.2 (37.9)	79.7 (40.5)	<0.001
Protein intake (g/day), mean (SD)	65.7 (22.2)	65.0 (23.2)	66.6 (22.3)	69.6 (25.6)	<0.001
Carbohydrate intake (g/day), mean (SD)	286.9 (100.9)	291.5 (101.1)	303.9 (98.3)	324.0 (110.2)	<0.001
Age (years), mean (SD)	50.3 (16.0)	52.2 (14.1)	51.3 (14.3)	48.9 (14.7)	<0.001
BMI (kg/m^2^), mean (SD)	23.4 (3.5)	23.5 (3.5)	23.3 (3.4)	22.9 (3.3)	<0.001
BMI status, *n* (%)					0.004
Underweight	222 (6.7%)	157 (6.1%)	98 (5.8%)	42 (6.3%)	
Normal	2102 (63.0%)	1612 (62.7%)	1091 (64.3%)	469 (70.2%)	
Overweight	859 (25.7%)	682 (26.5%)	454 (26.8%)	135 (20.2%)	
Obese	153 (4.6%)	120 (4.7%)	54 (3.2%)	22 (3.3%)	
Sex, *n* (%)					0.006
Men	1546 (45.6%)	1226 (46.8%)	852 (49.2%)	358 (52.0%)	
Women	1844 (54.4%)	1391 (53.2%)	881 (50.8%)	331 (48.0%)	
Income, *n* (%)					<0.001
Low	901 (26.9%)	753 (29.0%)	484 (28.4%)	226 (33.3%)	
Medium	1161 (34.7%)	757 (29.2%)	583 (34.2%)	242 (35.6%)	
High	1286 (38.4%)	1086 (41.8%)	638 (37.4%)	211 (31.1%)	
Education, *n* (%)					0.31
Low	1373 (40.5%)	1092 (41.8%)	730 (42.2%)	305 (44.4%)	
Medium	1183 (34.9%)	872 (33.4%)	586 (33.9%)	238 (34.6%)	
High	830 (24.5%)	647 (24.8%)	412 (23.8%)	144 (21.0%)	
Hypertension, *n* (%)	964 (28.6%)	765 (29.5%)	430 (25.1%)	135 (19.9%)	<0.001
Diabetes, *n* (%)	388 (11.4%)	305 (11.7%)	150 (8.7%)	61 (8.9%)	0.002
Urbanization, *n* (%)					<0.001
Low	514 (15.2%)	390 (14.9%)	236 (13.6%)	85 (12.3%)	
Medium	1014 (29.9%)	1015 (38.8%)	677 (39.1%)	322 (46.7%)	
High	1862 (54.9%)	1212 (46.3%)	820 (47.3%)	282 (40.9%)	
Smoking, *n* (%)					0.006
Non-smoker	2389 (70.5%)	1776 (67.9%)	1174 (67.8%)	456 (66.3%)	
Ex-smoker	126 (3.7%)	90 (3.4%)	51 (2.9%)	15 (2.2%)	
Current smoker	874 (25.8%)	749 (28.6%)	507 (29.3%)	217 (31.5%)	
High sensitivity CRP (mg/dL), mean (SD)	1.0 (0.0–2.0)	1.0 (0.0–2.0)	1.0 (0.0–2.0)	1.0 (0.0–2.0)	0.71
CKD, *n* (%)	445 (13.1%)	305 (11.7%)	207 (11.9%)	51 (7.4%)	<0.001
Physical activity (MET hour/week), mean (SD)	120.4 (105.1)	130.0 (112.6)	121.3 (104.5)	120.8 (106.2)	0.006

^1^ Data are presented as mean (SD) for continuous measures, and n (%) for categorical measures.

**Table 2:** Odds ratios (95% CI) for chronic kidney disease according to cumulative chili intake among Chinese adults (N = 8429) ^1^.

	None	1–20 g/day	20.1–50 g/day	≥50.1 g/day	*p* Value
	N = 3390	N = 2617	N = 1733	N = 689	
Model 1	1.00	0.82 (0.68–0.98)	0.97 (0.79–1.19)	0.63 (0.45–0.89)	0.057
Model 2	1.00	0.81 (0.66–0.99)	0.83 (0.66–1.05)	0.48 (0.33–0.71)	0.001
Model 3	1.00	0.82 (0.67–1.01)	0.83 (0.65–1.05)	0.51 (0.35–0.75)	0.001
Sensitivity analysis	1.00	0.78 (0.54–1.11)	0.73 (0.49–1.08)	0.47 (0.26–0.85)	0.012

^1^ Model 1 was adjusted for age in 2009, gender, intake of energy. Model 2 was further adjusted for education (low, medium and high), income, urbanization level (tertiles), smoking, alcohol drinking, physical activity, and dietary patterns (average scores between 1991 and 2009). Model 3 was further adjustment for overweight/obesity, hypertension, and diabetes. Sensitivity analysis was Model 3 including only those who attended all seven waves of the survey.

**Table 3:** Odds ratios (95% CI) for chronic kidney disease according to cumulative chili intake among Chinese adults by sociodemographic factors and health conditions (N = 8429) ^1^.

	None	1–20 g/day	20.1–50 g/day	≥50.1 g/day	*p* Value
Gender					
Men	1.00	0.74 (0.52–1.06)	0.74 (0.50–1.10)	0.47 (0.25–0.89)	0.920
Women	1.00	0.87 (0.67–1.13)	0.90 (0.66–1.21)	0.53 (0.32–0.87)	
Income					
Low	1.00	0.77 (0.53–1.11)	0.59 (0.38–0.90)	0.45 (0.24–0.85)	0.310
Medium	1.00	0.68 (0.44–1.03)	0.93 (0.61–1.42)	0.53 (0.29–0.99)	
High	1.00	1.05 (0.76–1.45)	1.14 (0.77–1.70)	0.51 (0.23–1.16)	
Urbanization					
Low	1.00	0.98 (0.50–1.90)	0.36 (0.14–0.94)	0.30 (0.09–1.05)	0.769
Medium	1.00	0.83 (0.57–1.22)	0.84 (0.56–1.26)	0.47 (0.26–0.85)	
High	1.00	0.82 (0.63–1.08)	0.93 (0.68–1.28)	0.62 (0.35–1.12)	
Overweight/obesity					
No	1.00	0.88 (0.68–1.12)	0.75 (0.56–1.00)	0.50 (0.32–0.78)	0.350
Yes	1.00	0.71 (0.49–1.04)	1.06 (0.70–1.61)	0.55 (0.25–1.23)	
Hypertension					
No	1.00	0.85 (0.64–1.13)	0.78 (0.57–1.06)	0.57 (0.36–0.91)	0.616
Yes	1.00	0.78 (0.57–1.06)	0.91 (0.63–1.32)	0.40 (0.20–0.80)	
Diabetes					
No	1.00	0.87 (0.69–1.09)	0.79 (0.61–1.02)	0.51 (0.33–0.77)	0.303
Yes	1.00	0.62 (0.37–1.03)	1.21 (0.64–2.26)	0.50 (0.18–1.35)	

^1^ Models adjusted for age in 2009, gender, intake of energy, education (low, medium, high), income (low, medium, high), urbanization level (tertiles), smoking, alcohol drinking, physical activity, dietary patterns (average scores between 1991 and 2009), overweight/obesity, hypertension, and diabetes. All the stratification variables were not adjusted in the corresponding models.

## Supplementary Materials

The following are available online at https://www.mdpi.com/2072-6643/11/12/2949/s1, Figure S1: Samples of fresh and dried chili in the Chinese market.
