# Supplementary figures and images for: Chili Intake Is Inversely Associated with Chronic Kidney Disease among Adults: A Population-Based Study
Source: Nutrients. 2019 Dec 4;11(12):2949. doi: 10.3390/nu11122949 (PMC6949978; doi:10.3390/nu11122949)

**Figure S1** Samples of fresh and dried chili in the Chinese market

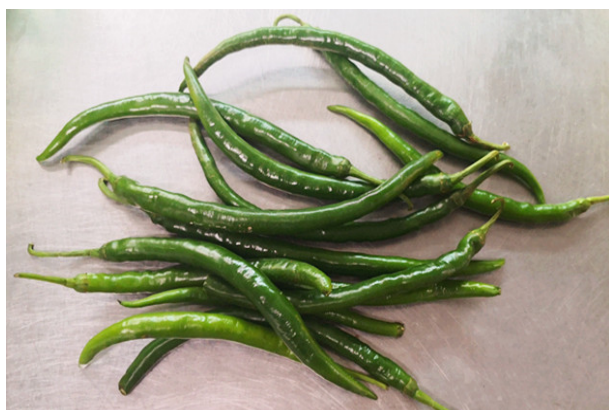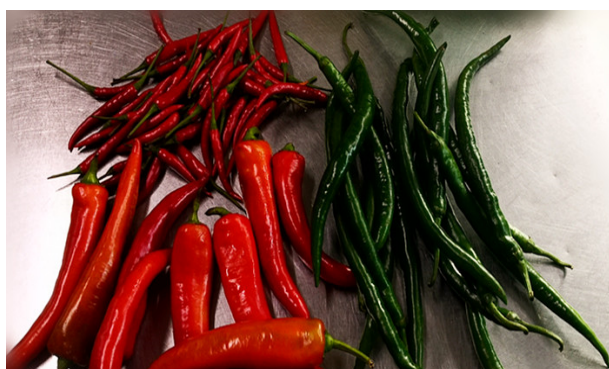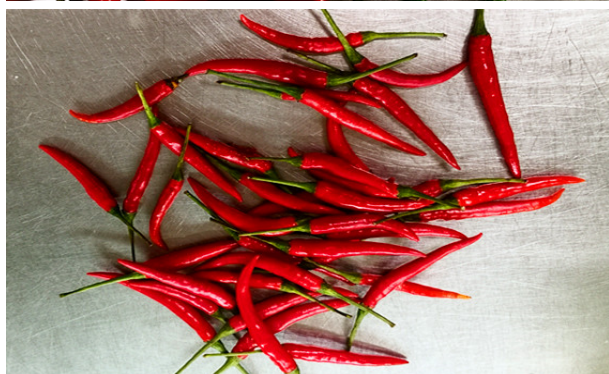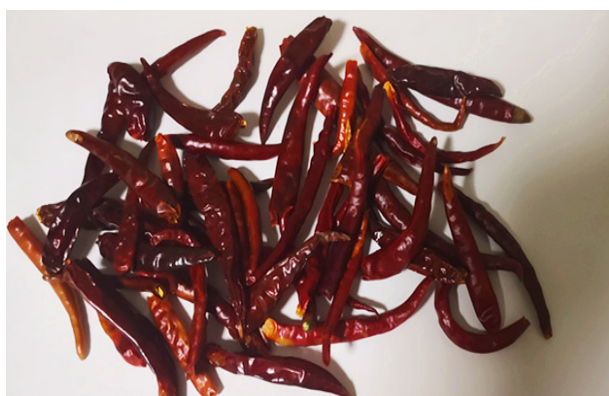

Supplement: Supplementary file 1 [file nutrients-11-02949-s001.pdf]
